# Physiologically Difficult Airway in the Patient with Severe Hypotension and Metabolic Acidosis

**DOI:** 10.1155/2020/8821827

**Published:** 2020-09-04

**Authors:** Joseph Capone, Vicko Gluncic, Anita Lukic, Kenneth D. Candido

**Affiliations:** ^1^Department of Anesthesia, Advocate Illinois Masonic Medical Center, 836 W Wellington Ave, Chicago, IL 60657, USA; ^2^Department of Anesthesia, Varazdin General Hospital, 1 I Mestrovica Street, Varazdin, HR 42000, Croatia; ^3^Department of Physiology, Nursing Studies, Bjelovar University of Applied Sciences, Eugen Kvaternik Square 4, Bjelovar, HR 43000, Croatia

## Abstract

The expertise to recognize and manage the difficult airway is essential in anesthesiology. Conventionally, this refers to anatomical concerns causing difficulties with facemask ventilation and/or with tracheal intubation. Severe derangements in patients' physiology can make induction and intubation likewise difficult, and approximately 30% of critically ill patients had cardiovascular collapse subsequently to intubation. We present the case of a 58-year-old male with a past medical history of type II diabetes and hypertension who presented with altered mental status due to severe metabolic acidosis with a pH of 6.8 on admission to the intensive care unit. The anesthesia team was called to urgently intubate the patient. Upon arrival, the patient was localizing to pain and was hypocapnic, tachycardic, and hypotensive despite ongoing therapy with norepinephrine, vasopressin, and bicarbonate drips. Bedside point-of-care ultrasound showed hyperdynamic left ventricle with no other abnormalities. The patient was induced with IV ketamine, and dissociation occurred with maintenance of spontaneous respirations, which was followed by laryngoscopy and intubation causing only minimal hemodynamic changes. The patient was subsequently dialyzed and treated supportively. He was discharged from the hospital two weeks later—neurologically intact and at his baseline. Combination of hypotension and severe metabolic acidosis is particularly a challenging setting for airway management and a major risk factor for adverse events, including cardiopulmonary arrest. Hemodynamically stable induction agents should be preferred. In addition, sustaining spontaneous ventilation and avoiding periods of apnea in the peri-intubation period is paramount—any buildup of CO_2_ could push a critically low pH even lower and cause cardiovascular collapse. Sympathomimetic properties of ketamine make this induction agent a particularly appealing choice in this setting. This case report further supports the concept that severe physiologic perturbations—in which conventional induction techniques are not feasible—should be included in the current definition of a difficult airway.

## 1. Introduction

The expertise to recognize and manage difficult airway is an essential skill in anesthesiology practice. Conventionally, this has referred to anatomical apprehensions causing difficulties with facemask ventilation and/or with tracheal intubation. However, severe derangements in patient physiology can make induction and intubation of critically ill patients just as perilous as anatomically difficult airway and therefore deserve the same level of consideration [1–5]. The term physiologically difficult airway refers to airway management in a critically ill patient with severely deranged physiology, which poses high risk for cardiopulmonary collapse and even arrest during or immediately after airway management [5]. The common physiologically difficult airways include settings of oxygen consumption increase, right ventricular failure, acidosis (metabolic), hypoxemia (saturation), and hypotension/hypovolemia (also memorized as mnemonic CRASH) [6] (Table 1). Other such examples include critical aortic stenosis, pulmonary hypertension, and cardiac tamponade [4–8]. Although approximately 30% of critically ill patients had cardiovascular collapse following intubation, there are limited data available on the physiologically difficult airway management approaches, especially when several conditions are concurrent [2–5].

Here, we present the case of combination of hypotension and severe metabolic acidosis, which is particularly a challenging setting for airway management and a major risk factor for adverse events, including cardiopulmonary arrest.

## 2. Case report

A 58-year-old male with a past medical history of type II diabetes and hypertension presented to the hospital with altered mental status and was found to have severe metabolic acidosis presumably due to metformin toxicity. He was admitted to the intensive care unit (ICU), where his mental status deteriorated, at which point the medical code team was called to intubate the patient. Upon arrival, the patient was localizing to pain, breathing 30 breaths per minute, with heart rate in the 110 s and blood pressure of 80/50 mmHg on norepinephrine, vasopressin, and bicarbonate drips. Arterial blood gas showed a pH of 6.8, pCO_2_ of 22 mmHg, pO_2_ of 134 mmHg on 3 L nasal cannula, undetectable HCO3, and lactate >24 mmol/L. Bedside echo showed a hyperdynamic left ventricle with no obvious regional wall motion abnormalities and no pericardial effusion.

The patient was preoxygenated with a nasal cannula and bag valve mask with O_2_ at 15 L/minute. A ketamine bolus of 140 mg (approximately 2 mg/kg) was given via the IV route that leads to patient dissociation but with the maintenance of spontaneous respirations. Following this, laryngoscopy was performed, and the patient was intubated with minimal change in hemodynamics.

Subsequently, he was connected to the ventilator and set on the assist control volume mode with a rate of 28 beats per minute, tidal volume of 450 mL, 4 cmH_2_O of positive end-expiratory pressure (PEEP), and 100% FiO_2_. The patient was subsequently treated with dialysis and supportive care. He was discharged from the medical ICU on hospital day 6 and discarded home on hospital day 14 at his baseline/when neurologically intact.

## 3. Discussion

A critically ill patient with hypotension and severe metabolic acidosis is particularly a challenging setting for airway management [4, 5], mainly due to the postintubation hypotension, which could even lead to cardiac arrest.

Hypotension during induction and intubation is a major risk factor for complications, including cardiopulmonary arrest, longer ICU stays, and increased mortality. In fact, hypotension and preinduction shock index (heart rate/systolic blood pressure) > 0.8 carry significant risk for postintubation hypotension and subsequent cardiac arrest [2, 5]. Focused assessed transthoracic echo (FATE) exam is a valuable tool when emergently called to manage critically ill patients with undifferentiated hypotension [5, 8, 9].

Patients with reduced venous return are especially prone to hypotension. These patients should be hemodynamically optimized prior to intubation with volume resuscitation if the patient is likely to be a volume responder, and hemodynamically stable induction agents should be used. If the patient is unresponsive to volume resuscitation, a norepinephrine infusion should be considered [4, 5, 9]. Finally, if resuscitation is not possible due to impending cardiopulmonary arrest in patients with severe shock, peripherally administered vasopressor boluses should be given to maintain blood pressure during intubation and resuscitation. Due to its inotropic effect, diluted epinephrine (10–50 *μ*g boluses/concentration of 10 *μ*g/mL) may be preferred in these situations [4, 5].

If hemodynamic stability cannot be reached before intubation, the choice of induction agents is critical for successful airway management in severely hypotensive patients. Propofol and benzodiazepines have a sympatholytic effect. This may cause decrease in vascular tone and myocardial depression and therefore further exacerbate peri-intubation hypotension. Furthermore, not only there are higher concentrations of propofol found in the brain of shocked patients, but in shocked patients, the brain becomes more sensitive to propofol also [10]. Therefore, the induction dose of propofol in shocked patients should be carefully titrated and reduced by 80–90% [10].

On the other hand, etomidate—which is a nonbenzodiazepine sedative—has been shown to be relatively hemodynamically neutral [4, 5], which means there is actually no need for dose reduction in shocked patients [11]. Ketamine is a particularly suitable agent in this situation due to sympathomimetic properties [3, 5, 12, 13]. In addition, some neuromuscular blocking agents have indirect cardiovascular effects through histamine release and parasympathetic activity which should also be taken into consideration [3–5, 12].

Therefore, in a severely hypotensive patient, preintubation fluid resuscitation and careful choice of induction agents are essential to ensure hemodynamic stability with induction and intubation [4, 5].

Deteriorating acidosis can cause seizure, coma, cardiac arrhythmia, and arrest [4, 5]. A recent study showed that mortality rates among ICU patients who presented with extreme acidosis (pH < 7.00) depend on the etiology of acidosis. Patients who suffered cardiac arrest prior to admission had a mortality of approximately 90%, while those who did not suffer had much lower mortality of approximately 57%. Regardless, the average Simplified Acute Physiology Score (SAPS) of the patients who did not suffer cardiac arrest in this study was still 82 (range: 69–93) which roughly carries much worse prognosis, i.e., predicted mortality of 75–90%. Therefore, in the absence of cardiac arrest, extreme acidotic patients should be aggressively treated in the ICU despite apparently “incorrectly” poor SAPS on ICU admission [1, 3].

If underlying cause of acidemia is respiratory acidosis, rapid improvement can be achieved by increasing alveolar ventilation. Interventions that increase the alveolar ventilation such as bag valve mask ventilation, NIPPV, or mechanical ventilation in most of the cases instantly decrease PaCO_2_ and correct respiratory acidosis [4, 5].

If metabolic acidosis is the underlying cause of acidemia, compensatory respiratory alkalosis from alveolar hyperventilation becomes critical for preservation of acid-base homeostasis. When hypocapnia develops due to a compensatory respiratory alkalosis, further hyperventilation results in subsequently smaller decreases in PaCO_2_ that eventually reaches a plateau. In this particular setting, the organic acid production requires alveolar ventilation that is difficult to achieve and patients subsequently develop profound acidemia. This is especially pertinent to severe metabolic acidosis from diseases such as diabetic ketoacidosis (DKA), salicylate toxicity, and severe lactic acidosis [3–5].

In patients with severe metabolic acidosis, whose high-minute ventilation requirement may not be met by the mechanical ventilator, intubation should be avoided or delayed, as long it is reasonable despite a critically low pH. In these cases, noninvasive positive pressure ventilation—measuring the patient's respiratory rate and tidal volumes—may provide an accurate estimate of the patient's intrinsic minute ventilation and adequately support hyperventilation until treatment of the underlying metabolic acidosis is initiated [3–5, 14].

If intubation of a patient with severe metabolic acidosis is necessary, preserving spontaneous respiration is critical both during intubation and with mechanical ventilation. Throughout induction and intubation, it is important to avoid even very brief periods of apnea as any additional CO_2_ accumulation can drive a critically low pH even further down and trigger cardiac arrest [3–5, 12, 14]. Transnasal humidified rapid-insufflation ventilatory exchange and similar methods that combine the benefits of apneic oxygenation with continuous positive airway pressure and gaseous exchange through flow-dependent dead-space flushing may be particularly useful in this case to increase apnea time [4, 14, 15]. In addition, rapid sequence intubation is objectively perilous in this setting, and if one is needed, short-acting neuromuscular blocker should be used [4, 5, 14].

Subsequently to intubation, a ventilator setting that allows the patient to maintain their own excessive minute ventilation should be selected so that their respiratory compensation can be sustained. Compensatory hyperventilation puts these patients at high risk of developing relative hypoventilation, flow starvation, patient-ventilator dyssynchrony, and further deteriorating acidosis. Therefore, emphasize is to optimize patient-ventilator synchrony and maintain high minute ventilation, especially in the spontaneously breathing patient [3–5]. Pressure-targeted modes, such as pressure support ventilation or pressure control, are particularly useful in this scenario as they allow the patient to set the rate and tidal volume received and maintain high-minute ventilation. Finally, particular attention should be paid to use sedative agents that do not affect the patient's respiratory drive, monitor for air trapping, given the high rates and tidal volumes, and monitor for respiratory muscle fatigue, as each of these can result in a loss of respiratory compensation [3–5, 14].

Similarly, in the case of acidosis, hemodynamic instability and hypotension caused by induction agents mentioned above could impair peripheral perfusion and worsen acidosis. Therefore, induction agents should be carefully selected and dose titrated.

In conclusion, this case report further supports the concept that patients with severe physiologic perturbations in whom conventional induction techniques are not feasible should be included in the current definition of a difficult airway [3, 4].

## Figures and Tables

**Table 1:** Physiologically difficult airway and the common disturbances, underlying conditions, and mitigations.

Disturbance	Underlying conditions	Consequences	Prevention/mitigation
Consumption increase	Sepsis Acute respiratory distress syndrome Excited delirium Thyrotoxicosis Pregnancy Children	Rapid desaturation during apnea	Thorough preoxygenation Minimal apnea time Adequate oxygenation Anemia correction

Right ventricular failure	Severe pulmonary arterial hypertension Massive pulmonary embolism	Right ventricle dilation and tricuspid regurgitation (especially after fluids administration) Hypercapnia Atelectasis Hypoxemia	Bedside cardiac ultrasound Right ventricle afterload reduction: pulmonary vasodilators Avoidance of hypoxia, hypercapnia, and acidosis Thorough preoxygenation Apneic oxygenation Careful fluid administration Vasopressors

Acidosis (metabolic)^*∗*^	Diabetic ketoacidosis Lactic acidosis Salicylate intoxication Severe sepsis Major trauma	Suppressed compensatory hyperventilation and worsened acidosis	(Avoid intubation) (Noninvasive positive pressure ventilation) Maintenance of spontaneous respiration during intubation Minimal apnea time Underlying cause treatment

Hypoxemia	Pulmonary diseases–pneumonia, acute respiratory distress syndrome, chronic obstructive pulmonary disease Pulmonary edema (cardiac, noncardiac)	Severe and rapid desaturation Hypoxic brain injury Cardiac dysrhythmias Cardiac arrest	Anxiolysis and analgesia Thorough preoxygenation Upright positioning Noninvasive positive pressure ventilation Apneic oxygenation Continuous positive airway pressure Awake intubation Anemia correction

Hypotension^*∗*^/hypovolemia	Volume depletion: hemorrhage and dehydration Vasoplegia: sepsis and anaphylaxis Capillary leak Cardiomyopathy	Severe sensitivity to induction agents Collapse following positive pressure ventilation Cardiac arrest	Preoxygenation Fluid resuscitation Use of hemodynamically neutral drugs Vasopressors Inotropes

^*∗*^Disturbances present in the patient presented in this case.

## Data Availability

The data (clinical/laboratory findings and medical knowledge) used to support the findings of this study are included within the article.
